# Understanding the Best Nutritional Management for Creutzfeldt–Jakob Disease Patients: A Comparison Between East Asian and Western Experiences

**DOI:** 10.3390/life14111496

**Published:** 2024-11-17

**Authors:** Alessia Perna, Massimo Santoro, Elisa Colaizzo

**Affiliations:** 1 Center for Neuromuscular and Neurological Rare Diseases, San Camillo Forlanini Hospital, 00152 Rome, Italy; 2 Division of Biotechnologies, Italian National Agency for New Technologies, Energy and Sustainable Development (ENEA), 00123 Rome, Italy; massimo.santoro@enea.it; 3 Department of Public Health and Infectious Disease, University of Rome “Sapienza”, 00185 Rome, Italy; elisa.colaizzo@uniroma1.it

**Keywords:** Creutzfeldt–Jakob disease, prion disease, artificial feeding, tube feed, gastrostomy, nutrition management

## Abstract

(1) Background: Creutzfeldt–Jakob disease (CJD) is a rare and fatal neurodegenerative disorder caused by the accumulation of an altered prion protein, which usually leads to death within one year after clinical onset. CJD patients usually present with rapid cognitive impairment associated with declines in cerebellar, motor, visual, behavioral, and swallowing functions. Moreover, CJD patients lose their ability to eat and take medications orally very early on in treatment; nevertheless, there are no specific nutritional guidelines for this disease shared worldwide. (2) Methods: This review aims to describe the nutritional outcomes of CJD patients in Western countries to compare them with those described in East Asian countries and then aims to explore the most recent trends in the nutritional management of CJD patients, including some dietary compounds that present neuroprotective effects. (3) Results: In Japan’s, Taiwan’s, and China’s healthcare systems, CJD patients receive intensive life-sustaining treatment that prolongs their survival (i.e., artificial feeding); conversely, in Western countries, intensive life-sustaining treatments like tube feeding are not commonly provided to CJD patients. (4) Conclusions: It is difficult to pinpoint the reasons for these discrepancies around CJD palliative care supply, but it is clear that specific nutritional guidelines may directly improve the nutritional management of CJD patients and thus allow their families and caregivers to ensure the best end-of-life care for these patients.

## 1. Introduction

Prion diseases are a heterogeneous group of neurodegenerative diseases caused by the brain accumulation and propagation of misfolded prion protein (PrP) isoforms, specifically called prions (PrP^Sc^). Prions are non-conventional pathogenic proteins that are caused by the conformational change of a protein normally present in our body called the prion protein (PrPC). Creutzfeldt–Jakob disease (CJD) can be sporadic, genetic, iatrogenic, or variant [1]. In humans, the most common form is sporadic CJD (sCJD), which occurs worldwide, with an incidence rate of approximately one to two cases per million people and a mean age of onset between 57 and 62 years, without gender predilection [2]. The median survival time for individuals with sCJD is approximately 5 months from the onset of symptoms [3]. Clinically, sCJD is characterized by rapidly progressive dementia, associated with ataxia, myoclonus, or other neurologic signs, leading to akinetic mutism [2]. More rarely, the disease may start with a focal neurologic deficit, such as progressive hemiparesis, extrapyramidal syndrome, status epilepticus, or myoclonus [4]. Human-to-human transmission of these diseases is due to iatrogenic exposures; in particular, the zoonotic forms of prion disease are linked to bovine diseases. The genetic forms are associated with mutations in the prion protein gene (*PRNP*) in genetic CJD, fatal family insomnia (FFI), and Gerstmann–Sträussler–Scheinker syndrome (GSS). Iatrogenic CJD (iCJD) is caused by accidental transmission. Clinical tools for diagnosis comprise electroencephalogram (EEG), brain imaging, and cerebrospinal fluid (CSF) findings, which allow for the detection of abnormally folded prion proteins. 

CNS involvement is the core manifestation of CJD: cognitive, behavioral, and affective symptoms influence not only the quality of life but also the prognosis of CJD patients. In fact, CJD patients can show symptoms of depression, personality change, insomnia, poor short-term memory, agitation, and hallucinations very early, from which their caregivers can experience terrible frustration and difficulty in accepting the course of the disease. Nowadays, palliative care specialists play a fundamental role both in the management of all these rapidly evolving complications for the people affected by CJD as well as in supporting their caregivers to better deal with this burden of suffering due to the patient’s rapid clinical decline [4].

However, among the still-unsolved issues pointed out by extensive and updated reviews on disease management for end-stage neurological disorders, i.e., CJD, AD (Alzheimer’s Disease), and ALS (Amyotrophic Lateral Sclerosis), research is being carried out regarding the role of nutritional assessment and management in prolonging the survival of CJD patients, which also serves to allow them to be enrolled in future therapeutic trials [3].

This crucial issue has particular relevance when we consider that, at present, there is no effective treatment available for CJD, which is always fatal, and so treatment is merely supportive and can be negatively affected by caregivers’ scarce compliance to medical recommendations. Conversely, nutrition maintenance can be considered as an outstanding factor in well-being, even in short-life-expectancy patients, such as CJD patients. This review aims to describe and interpret studies on nutritional outcomes of CJD patients in Western countries and compare them with those described for patients in East Asian countries in order to decipher the best impact on healthcare needs and quality of life.

## 2. Materials and Methods

**Figure 1:**
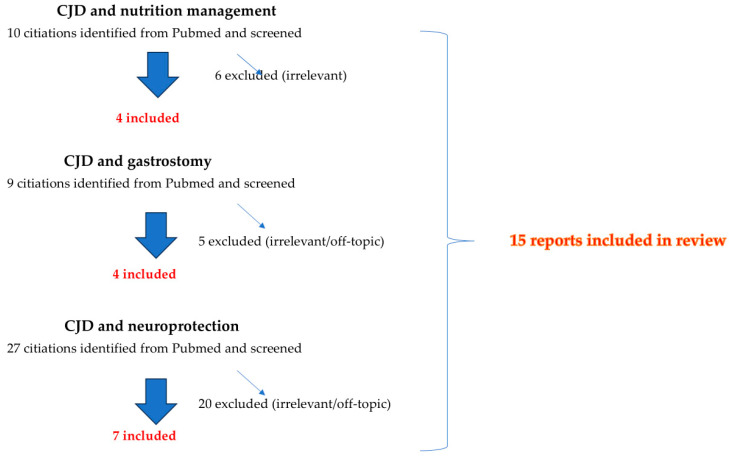
Study selection flow-chart.

Our mini-review includes research studies from 2000 to 2024 selected through the PubMed database, with a variable combination of “Creutzfeldt-Jakob disease” with “nutrition management”, “artificial feeding”, “tube feed”, “gastrostomy”, “neuroprotective compound”, and “nutritional guidelines” as keywords. Consensus statement articles were not included (Figure 1).

## 3. Results

### 3.1. CNS and Nutrition

Recent research suggests that food insecurity is associated with worse cognitive functioning [5], and a loss of weight is considered to be an independent poor prognostic factor in neurodegenerative diseases, including ALS patients; ALS patients with mild obesity presented longer survival times than underweight patients. These results highlight the relevance of nutritional issues in cognitive diseases, especially in which dysphagia, mood disorders, or severe upper-limb hyposthenia prevent autonomous feeding. Moreover, an interesting study underlines the important role of diet/nutrition in decreasing dementia and depression risk in individuals with a predisposition to developing neurodegenerative diseases [6].

### 3.2. Dysphagia

**Figure 2:**
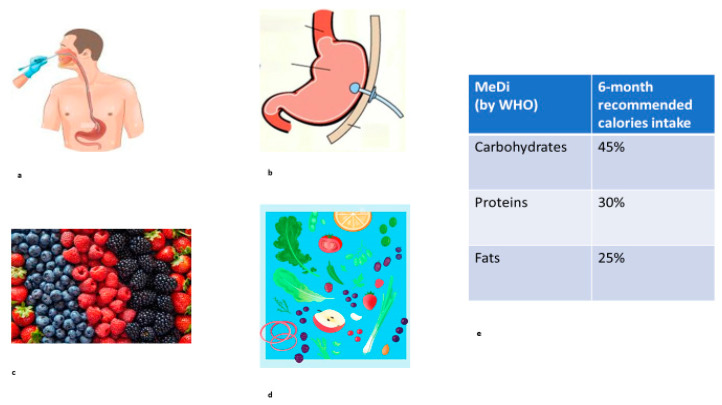
Tools of nutrition management in CJD patients: (**a**) nasal tube placement; (**b**) percutaneous endoscopic gastrostomy tube placement; (**c**) foods containing anthocyanins; (**d**) foods containing flavonoids; (**e**) high-calorie dietary supplements according to the MeDi, the Mediterranean diet, as recommended by the WHO (World Health Organization) [19].

In dementia management, regular swallowing assessment is strictly suggested to identify an early impairment and prevent malnutrition leading to weight loss. In the literature, swallowing ability in long-term survivors of Creutzfeldt–Jakob disease (CJD) has not been disclosed: in particular, a sporadic CJD case with MM2-cortical-type sCJD (MM2C-type sCJD) characterized by slowly progressive pharyngeal swallowing disfunction was reported. [7] Slower eating and chewing, associated with avoiding food and medications orally, and coughing during eating are common symptoms of the moderate and advanced stages of prion disease. What is more, a swallowing impairment can worsen respiratory complications and pulmonary aspiration. Consequently, dysphagia management can have a positive impact on QOL (quality of life) in CJD patients, and consists of the following:

- Safe behavioral interventions, such as adjusting food and fluid consistency, upright positioning for improving deglutition, and preventing aspiration;
- Dietary modifications, such as high-calorie feeding to contrast loss of weight;
- Nasal tube then/or percutaneous endoscopic gastrostomy (PEG) tube placement to elude an oral feeding deficit (Figure 2a,b).

The positioning of devices used for artificial feeding in patients with prion disease requires great care. In fact, endoscopic instruments used on CJD patients must be decontaminated by appropriately trained personnel and include the adoption of containment measures to minimize the accidental dispersion of potentially infected materials and liquids. It is always advisable to avoid recycling the detergents or disinfecting liquids and brushes used in each endoscopic procedure [8]. Percutaneous endoscopic gastrostomy (PEG) tube placement is commonly considered the preferred alternative to nasogastric feeding to provide long-term enteral feeding in patients who develop severe dysphagia [9]. It is recommended as the safest procedure, particularly in late-stage dysphagia due to neurological disorders such as ALS, dementia, stroke, and Multiple Sclerosis with a high risk of protein-calorie malnutrition and pneumonia aspiration. The PEG tube is inserted into the stomach through the anterior abdominal wall during gastroscopy for placement. Since its introduction in the 1980s by Gauderer, it stands out as a less-invasive nutritional option and is safer than surgical or radiological insertion techniques [10].

There are not many reports that have investigated in great detail what the key factor contributing to prolonging survival of CJD patients is.

In the literature, the total disease duration, which is obviously increased in slowly evolving CJD forms, is significantly correlated with the time from clinical onset of myoclonic jerks, the recognition of periodic sharp-wave complexes on electroencephalograms, and evolution to the akinetic mutism state.

Our interest in the nutritional management of CJD patients arises from the in-depth comparison of the clinical features and duration of prion disease in Western countries and Eastern ones. The Western literature about CJD clinical findings is based on data provided by the EUROCJD (European Creutzfeldt–Jakob Disease Surveillance Network), which includes since 1993 Australia, Austria, Canada, France, Germany, Italy, The Netherlands, Slovakia, Spain, Switzerland, and the United Kingdom, and by the NEUROCJD (Extended European Collaborative Study Group of CJD) comprising Belgium, Denmark, Finland, Greece, Iceland, Ireland, Israel, Norway, and Portugal since 1998. The Eastern studies about CJD clinical trends do not derive from international surveillance systems, but from national programs, as in the case of Japan, where prion diseases have been classified in the Specific Intractable Diseases Treatment Program since 1997 [11].

Pursuant to Western countries (Table 1), in 1999, Parchi et al. found that for 300 sporadic CJD patients, studied between 1968 and 1998 from North America (United States and Canada) and Europe (France, Italy, United Kingdom, Finland, and Denmark), the shortest average duration of symptoms was 3.9 months (range 1–18) for (203) patients homozygous for methionine/methionine (MM) at codon 129 in the *PRNP*, and the longest duration was 17.1 months (range 5–72) for (27) patients with the methionine/valine (MV) polymorphism [12].

In 2004, Pocchiari et al. carried out a collaborative study to define the predictors of survival in a large cohort of CJD patients, including 2304 sporadic cases, 106 iatrogenic, 86 variant, and 278 genetic (24 with Gerstmann–Sträussler–Scheinker syndrome and 41 affected by fatal familial insomnia). They were collected by the EUROCJD from 1993 to 2000. They confirmed the results reported by Parchi et al.: sCJD had a median survival time of 5 months. In particular, the longer duration of life correlated with younger age at onset, female gender, codon 129 heterozigosity, and the presence of the CSF 14-3-3 protein and type 2a protein. Conversely, the median clinical duration was 4 months for genetic CJD patients, shorter in males than in females and significantly shorter in V210I patients with late age at onset [3].

Lastly, in 2006, Collins et al. reviewed all 2451 sCJD patients that died between 1992 and 2002 in the EUROCJD countries and confirmed the same illness duration [13]. Unfortunately, in all these collaborative studies, CJD survival duration was never correlated with the nutritional setting.

Pursuant to East Asian countries (Table 2), on the contrary, we documented single case reports or small series reported to the national CJD Surveillance Committee in reference to disease duration and its relationship with nourishment.

In 2012, Iwasaki et al. described 2 cases of gCJD (one Gerstmann–Sträussler–Scheinker syndrome P102 L and one V180I) and 10 cases of sporadic CJD (7 MM1-type definite, 3 probable) that were hospitalized for a total of 3968 days in the akinetic mutism state. In all these cases, parenteral nutrition, symptomatic therapies, and antibiotic drugs were administered by a nasal tube. In the nine definite CJD cases, the median period from the akinetic mutism state to death was 22 months (average: 27.0 ± 23.3 months, range: 3–80 months), and the median total disease duration was 27 months (average: 34.2 ± 30.1 months, range: 5–102 months) [14].

In 2015, Iwasaki et al. assessed the average total disease duration for 26 men and 25 women affected by MM1-type sCJD and found that it was 12.3 ± 9.6 months (median: 9 months, range: 1–32 months), with no significant difference between men and women. In particular, they found that the average total disease duration was 4.0 ± 1.6 months in non-tube-fed patients as compared to tube-fed patients, in whom it was 16.1 ± 9.4 months; the difference between the two cohort’s durations was statistically significant. Notably, they reported that in total, three gastrostomies were performed as well [15].

To our knowledge, in a vast Chinese case history [16], Hsieh et al. retrospectively reviewed the medical charts of 30 patients diagnosed with probable sCJD in a medical center in Taiwan from 2002 to 2017 to determine if there are predictive factors for early NG tube feeding. NG tube insertion was performed on average 46.4 days after disease onset. Moreover, they noticed that myoclonus and detectable CSF 14-3-3 protein are associated with rapid deterioration of the patient’s condition and can be predictable of need for enteral nutrition.

To note, an interesting study edited by the Japanese Surveillance Committee suggested that for 1128 sporadic and genetic CJD patients, observed from 1999 to 2008, mean disease duration was 17.4 months (range: 1–143) [11].

Finally, Hayashi et al. described an extreme case of V180I gCJD: a 78-year-old Japanese woman presenting with pseudobulbar paralysis. She reached the akinetic mutism state 14 months after disease onset, and then she underwent tube feeding and survived for about another 50 months [7].

All in all, these similar data lead us to consider supportive care such as parenteral nutrition as decisive in improving quality of life and prolonging survival, both in the genetic and in the sporadic forms of the disease.

Recently, Kunieda et al. [17] described a 69-year-old woman with V180I genetic CJD (gCJD). Because of worsening dysphagia, she received percutaneous gastrostomy 42 months after the onset. In this case, a serial pharyngeal swallowing evaluation by videofluoroscopic examination (VF) highlighted that this function was preserved until 72 months after onset. MRI excluded any atrophy of the brainstem, suggesting that the dysphagia in a long-term survivor of V180I gCJD is rather caused by a bilateral cortico-bulbar tract disturbance. Notably, to better understand dysphagia in V180I gCJD patients, we also reviewed articles that described nutritional administration methods applied to ten patients, including their novel case. Six patients of this small cohort (60%) continued orally intaking food for an average of 16.6 ± 3.3 months despite deteriorating to the state of akinetic mutism. It is worth noting that two CJD patients in particular, including the proband, showed prolonged oral feeding of more than five years.

Lastly, Iwasaki et al. [18] described an autopsied case of a Japanese woman with Gerstmann–Sträussler–Scheinker disease (GSS) presenting with a rapidly progressive clinical course. Disease onset occurred at the age of 54 with dementia and gait disturbance; eleven months after, she reached the akinetic mutism state, and then nasal tube feeding was introduced and continued for 62 months.

**Table 2:** East Asian studies on average disease duration in East Asian single CJD case reports or small series in relation to nutritional management.

Eastern Study	CJD Patients	Average Disease Duration Months	Nasal Tube	Median Period Akinetic State–Death	Gastrostomy
Iwasaki et al. [14]	2 gCJD 10 sCJD	27	Yes	22	
Iwasaki et al. [15]	51 sCJD	9			3
Nagoshi et al. [11]	1128 sCJD and gCJD	17.4			
Hayashi et al. [7]	1 gCJD	64	Yes	50	
Kunieda et al. [17]	1 gCJD	72		42	Yes
Iwasaki et al. [18]	1 gCJD	73	Yes	11	

### 3.3. Tools of Nutrition Management

Nutrition maintenance is a crucial factor in patients with CJD, in whom swallowing impairment, anorexia, and loss of weight create a dead-end cycle of increased frailty. In this light, growing evidence concludes that it is challenging to support body weight gain in dependent elderly adults by high-calorie dietary supplements according to the MeDi, the Mediterranean diet, as recommended by the WHO (World Health Organization) [19]. In particular, for patients with dementia and frailty, such as CJD, it has been recommended to intake 500 kcal for 6 months, comprising approximately 45% carbohydrates, 30% proteins, and 25% fats that provide an enhanced level of vitamin E, B12, C, folate, zinc, and copper (for 12 months, 6 mg of vitamin B_12_ and 400 mg of folic acid have been recommended) [20] (Figure 2e). In this regard, it is known that supplements containing B and D vitamins and minerals might be beneficial in sustaining cognitive function and also that homocysteine levels can increase in various forms of dementia [21]. As described by Iwasaki et al. [22], tube-fed patients can be continuously administered 800–1000 kcal/day and checked day by day regarding idratation and calorie intake.

### 3.4. Neuroprotection by Nutrition in CJD

Above all, nutritional choices may also provide specific neuroprotective effects, although prion diseases are invariably fatal. Due to their potent anti-oxidant action, anthocyanins (ACNs), which are polyphenolic derivatives of the anthocyanidin flavonoid group, have been largely evaluated as potential remedies against oxidative-stress-associated conditions, such as neurodegeneration, because they are able to penetrate the Blood–Brain Barrier (BBB) [23]. In a recent study, Christoudia et al. demonstrated the ability of Oenin and Myrtillin, two of the most common anthocyanins, to de-aggregate pre-existing PrP^Sc^ fibrils and to inhibit de novo PrP^Sc^ fibrillation. Most of all, they acted as potent neuroprotective agents, decreasing ROS (reactive oxygen species) levels through the activation of the Keap1-Nrf2 pathway [24] (Figure 2c).

Interestingly, Glykofridi et al. investigated the properties of some novel flavonoids, rhenium–tricarbonyl complexes of 3,3′,4′,5,7-pentahydroxyflavone (quercetin), 3,7,4′-trihydroxyflavone (resokaempferol), 5,7-dihydroxyflavone (chrysin), and 4′,5,7-trihydroxyflavonone (naringenin), as potent anti-PrP agents. These flavonoids are natural polyphenolic substances ubiquitous in vegetables, fruits, tea, and other plants. In addition to their synthetic derivatives, they exhibit anti-oxidative capacities as well as inhibition of toxic β-amyloid formation; therefore, they are gaining an attractive role as therapeutic agents against neurodegeneration. In this study, the authors found that all these substances and their respective re-complexes, tested in vitro by performing the real-time quaking-induced conversion assay with recombinant PrP seeded with cerebrospinal fluid from CJD patients, blocked de novo abnormal formation and aggregation of the amyloid-like abnormal prion protein PrP^Sc^ [25] (Figure 2d).

Of the numerous natural dietary substances screened, an ingredient derived from the root of Curcuma Longa, curcumin, may potentially play a potential neuroprotective effect mediated by modifying the PI3K/AKT signaling pathway in dementia and neurodegenerative disorders [26].

## 4. Discussion

Currently, the insertion of an NG tube is indicated in many survivors of neurodegenerative diseases characterized by swallowing disorders: Alzheimer’s Disease, CJD, and ALS. In contrast to akinetic mutism, neurologists and physicians can recognize patients with severe neurodegenerative diseases by inserting an NG tube. Although the prolonged survival of patients with NG tube feeding may be affected by the presence of comorbidities such as aspiration pneumonia or other infectious diseases or prolonged hospitalization, it was found that the survival of CJD patients who received tube feeding after reaching the state of akinetic mutism, which is considered a neurological symptom of sCJD endpoint, is increased according to Nakatani et al. [27]. Furthermore, the presence of myoclonus, along with increased 14-3-3 protein in the CSF of CJD patients, could be considered as a predictive marker for the disease’s prognosis. In the end, the beginning of artificial feeding indicates an early irreversible dependence in daily activities.

To our knowledge, there are unfortunately no other reports detecting the efficacy of tube feeding or gastrostomy in cases of prion disease among North American and European CJD patients [28]. On the other hand, East Asian studies present some limitations: they are often based on single case reports or small case series, and the neuropathological findings often are unavailable because these patients are still alive, necessitating further evaluation of additional cases. These studies often lack longitudinal data and, most importantly, are not part of an International Disease Surveillance Network like the European one.

However, in Japan’s, Taiwan’s, and China’s cultures, in well-organized healthcare systems that include a health insurance system, the majority of patients undergo intensive life-sustaining treatment that prolongs their survival, and some even continue the treatment even if the advanced neurological disorder is irreversible [13].

Differently, in Western countries, due to financial and ethical findings, intensive life-sustaining treatments like tube feeding are not commonly provided to patients with fatal neurologic conditions, such as prion disease. The predictability of death may influence the ability of physicians to identify palliative situations and facilitate their engagement in end-of-life care planning [29].

For instance, in the USA, all the restrictions on coverage of expensive medications or treatments intended to prolong life can delay hospice admission, particularly in cases with a poor prognosis [11].

The comparative aspect of this review is limited, as it draws on results from different global regions over a span of 20 years, with varying cultural, ethical, and legal frameworks. It is difficult to disentangle the reasons for discrepancies about palliative care supply [26]. However, the results related to nutrition outcomes and recommendations for CJD patients indicate significant differences in how health and end-of-life issues are managed in Eastern versus Western countries.

Interestingly, in 2018, the European Society for Clinical Nutrition and Metabolism (ESPEN) published guidelines on clinical nutrition regarding some neurological diseases [30]: Amyotrophic Lateral Sclerosis, Parkinson’s Disease, Multiple Sclerosis, and stroke. Their guideline standard operating procedures (SOPs) were based on the methodology provided by the Association of Scientific Medical Societies of Germany (AWMF), the Scottish Intercollegiate Guidelines Network (SIGN), and the Centre for Evidence-based Medicine at the University of Oxford. In particular, the ESPEN recommended the following:

- ALS patients: regular assessment of BMI (body mass index), weight loss over time, lipid status, and a body composition analysis using DEXA (Dual-Energy X-ray Absorptiometry) or BIA (Bioelectrical Impedance Analysis) at diagnosis and every 3 months during follow-up in order to screen malnutrition and plan for nutritional supplementation and enteral feeding/gastrostomy.
- PD patients: routine monitoring of dysphagia during the ON-phase in the advanced phases along with supplementation of vitamin D, vitamin B12, and folic acid throughout the disease course.
- MS patients: early screening for swallowing disfunction, especially in those with cerebellar involvement, and the initiation of enteral nutrition as needed.
- Stroke patients: during the acute phase, there is a high risk of malnutrition, aspiration pneumonia, and dehydration, so that the enteral feeding is often necessary, even if the condition is not irreversible.

Nevertheless, numerous neurological diseases, including rare ones such as CJD, may result in swallowing impairment and consequently malnutrition. Therefore, it is hoped that ESPEN also draws up nutritional guidelines for CJD patients.

It is strictly urgent to share healthcare measures for the management of CJD patients that can be broadly used worldwide to allow comparisons and to share decision-making highlights [31]. Rising healthcare costs, especially in chronic diseases, are further exacerbated by the current global healthcare labor shortage. However, some countries have initiated aging policies focused on promoting care in aging populations: in Arab regions [32] and in China [33], approaches based on supporting basic well-being for the elderly have been undertaken, in contrast to Latin American countries, where the predominant healthcare policy is mainly inspired by a charity-based principle to restrain poverty [30].

Nevertheless, it is worth noting that the EUROCJD and NEUROCJD have effectively demonstrated that a rapid and early diagnosis enables combining behavioral approaches, pharmacological therapies, and nutritional measures with supportive care, public health interventions, and clinical trial recruitment [4].

## 5. Conclusions

The medical and economic cost of caring for elderly patients who are frail and demented, including CJD patients, is one of the most intriguing challenges of modern healthcare. There are currently numerous potential compensatory interventions to support the clinical and nutritional management of CJD-affected patients. Therefore, it is essential to start now by developing cost-effective studies that can be longitudinally applied across cultures to identify and implement the most effective strategies and biomarkers.

Based on a comparison between Western and East Asian experiences, the following are strongly recommended for CJD patients:

- A rigorous monitoring of swallowing function and weight loss to prevent malnutrition;
- Long-term tube feeding when patients deteriorate to an akinetic mutism state or exhibit myoclonus along with elevated 14-3-3 protein levels in the CSF;
- A nutritional intake of 500–1000 kcal/day, comprising 45% carbohydrates, 30% proteins, and 25% fats with 6 mg of vitamin B12 and 400 mg of folic acid;
- Evaluation of gastrostomy, with comprehensive informed consent from the patient’s family, particularly when prolonged nasal tube feeding leads to complications such as nasal wing erosion and aspiration pneumonia;
- Supplementation with ACNs, novel flavonoids, and curcumin.

Therefore, it is clear that further specific nutritional guidelines may directly improve not only the palliative care of CJD patients but also can ensure that the needs of their families and caregivers are really met and supported, especially because they are all experiencing this short life expectancy. However, the integration of palliative care into neurological services is relatively recent, which means that training specialists and educators in neuropalliative care will take time and importantly require a shift towards a more open-minded approach.

## Figures and Tables

**Table 1:** Western studies on average duration of symptoms in three large cohorts of North American and European sCJD patients between 1968 and 2002.

Western Study	CJD Patients	Average Duration Disease Months	Period
Parchi et al. [12]	300 sCJD	10.5	1968–1998
Pocchiari et al. [3]	2304 sCJD	5	1993–2000
Collins et al. [13]	2451 sCJD	5	1992–2000

## Abbreviations

PrP: prion protein; PrP^Sc^: isoforms of prion proteins; CJD, Creutzfeldt–Jakob disease; sCJD: sporadic CJD; PRNP: prion protein gene; FFI: fatal family insomnia; GSS: Gerstmann–Sträussler–Scheinker syndrome; iCJD: iatrogenic CJD; AD: Alzheimer’s Disease; ALS: Amyotrophic Lateral Sclerosis; QOL: quality of life; PEG, percutaneous endoscopic gastrostomy; NG: nasogastric tube; gCJD, genetic CJD; VF: videofluoroscopic examination; WHO: World Health Organization; ACNs: anthocyanins; BBB: Blood–Brain Barrier; ROS: reactive oxygen species; USA: United States of America; SOP: standard operating procedures; BMI: body mass index; DEXA: Dual-Energy X-ray Absorptiometry; BIA: Bioelectrical Impedance Analysis; CSF: cerebrospinal fluid.
